# Paternal personality and social status influence offspring activity in zebrafish

**DOI:** 10.1186/s12862-017-1005-0

**Published:** 2017-07-03

**Authors:** Susanne Zajitschek, James E. Herbert-Read, Nasir M. Abbasi, Felix Zajitschek, Simone Immler

**Affiliations:** 1 School of Biological Sciences, University of East Anglia, Norwich Research Park, NR4 7TJ Norwich, UK; 2 Department of Ecology and Genetics, Uppsala University, Norbyvägen 18D, 752 36 Uppsala, Sweden; 3 Doñana Biological Station EBD-CSIC, C/Americo Vespucio s/n, 41092, Isla de la Cartuja, Sevilla, Spain; 4 Department of Mathematics, Uppsala University, Lägerhyddsvägen 1, 751 06 Uppsala, Sweden; 5 School of Biological Sciences, Monash University, Building 18, Clayton, VIC 3800 Australia; 6 Department of Zoology, Stockholm University, 10691 Stockholm, Sweden

**Keywords:** Behavioural syndrome, Boldness, Context-dependence, Dominance, Sperm trait, Transgenerational effects

## Abstract

**Background:**

Evidence for the transmission of non-genetic information from father to offspring is rapidly accumulating. While the impact of chemical and physical factors such as toxins or diet on the fitness of the parents and their offspring have been studied extensively, the importance of behavioural and social circumstances has only recently been recognised. Behavioural traits such as personality characteristics can be relatively stable, and partly comprise a genetic component but we know little about the non-genetic transmission of plastic behavioural traits from parents to offspring. We investigated the relative effect of personality and of social dominance as indicators at the opposite ends of the plasticity range on offspring behaviour in the zebrafish (*Danio rerio*). We assessed male boldness, a behavioural trait that has previously been shown previously to possess genetic underpinnings, and experimentally manipulated male social status to assess the association between the two types of behaviour and their correlation with offspring activity.

**Results:**

We found a clear interaction between the relatively stable and putative genetic effects based on inherited differences in personality and the experimentally induced epigenetic effects from changes in the social status of the father on offspring activity.

**Conclusions:**

Our study shows that offspring behaviour is determined by a combination of paternal personality traits and on-genetic effects derived from the social status of the father.

**Electronic supplementary material:**

The online version of this article (doi:10.1186/s12862-017-1005-0) contains supplementary material, which is available to authorized users.

## Background

Evidence for the importance of transgenerational non-genetic effects in ecology and evolution is rapidly mounting [1, 2]. Environmental conditions experienced by females have long been known to affect offspring fitness [3–5] and more recently, similar transgenerational effects of conditions experienced by males have been established [6]. While the effect of environmental factors such as stress and nutrition on parent and offspring condition have received substantial attention (e.g. [2]), we are only starting to understand how social and behavioural patterns and conditions are transmitted from parents to offspring. A recent study in the house mouse *Mus musculus* for example showed that olfactory conditioning in juvenile males led to a heightened behavioural sensitivity to that same odour in their F1 and F2 offspring [7]. This striking result suggests that even events limited in time may have profound effects on behavioural patterns in the next generation. In order to fully understand how behavioural traits are inherited, it therefore is necessary to simultaneously evaluate the relative importance of behavioural patterns that may have a genetic underpinning and non-genetic (or epigenetic) effects. The aim of the present study was to assess how a relatively stable behavioural trait that has been shown to be heritable (boldness) is affected by varying male social status prior to siring offspring and how these traits in turn affect the behaviour and performance of the offspring.

The non-genetic transmission of behavioural patterns from parents to offspring and more specifically how behavioural reactions to stimuli experienced by parents may affect the behaviour of the offspring is receiving increasing attention [8, 9]. A study in house mice showed that exposure to aggressive encounters resulting in chronic defeat stress in males led to increased depression and anxiety-like phenotypes in their offspring [8]. Similarly, a lack of early handling experience influenced the social behaviour of prairie voles *Microtus ochrogaster* and led to reduced participation in alloparenting in the offspring of the following two generations [10]. But also more subtle factors, such as the composition of the social environment experienced by males during early development and prior to mating have been found to affect a wide range of traits in the next generation [9, 11, 12]. In stalk-legged flies *Telostylinus angusticollis* for example, males reared in a mixed sex and nutrient rich environment sired offspring that were larger and more viable than offspring of fathers raised on nutrient poor diets, but only when these had been kept in same-sex groups before mating [11]. Moreover, in the zebrafish *Danio rerio*, differences in male-male competition two weeks prior to mating affected the speed of hatching and led to differential survival in the offspring [12].

Personality traits that are characterized by individual differences in activity, sociability or boldness are supposed to be relatively stable within an individual over a range of different environments and over time [13, 14]. In fact, personality traits such as exploratory behaviour in great tits (*Parus major*, [15]), antipredator behaviour in Alpine swift (*Apus melba*, [16]) and dominance in chimpanzees (*Pan trolodytes*, [17]) as well as aggressiveness and boldness in zebrafish [18] are to some degree heritable, suggesting a genetic underpinning. Nevertheless, personality traits appear to retain high levels of context-dependent plasticity (but see [19]). Social context and group composition may play a major role in determining individual behaviour (e.g. [19, 20]). In perch (*Perca fluviatilis*, [21]), individuals classified as shy in random groupings are more likely to exhibit bold behaviour when grouped only with other shy individuals, highlighting the importance of social context. Furthermore, in domestic fowl *Gallus gallus domesticus*, personality was found to change with experimental alteration of social rank [22] illustrating the potential association between social hierarchy and behaviour. In contrast, in zebrafish, innate personality traits were found to predict future dominance status [23], albeit this was done without a priori knowledge of previous social rank. Importantly, both personality ([24], meta-analysis: [25]) and social status have been found to be linked to reproductive fitness in a range of species [26] including the zebrafish [27].

Even though personality may be indicative of social status (or vice versa) in some circumstances, the fact that status can easily be manipulated while personality traits are thought to be stable suggests that the interaction between the two is more complex than assumed so far. Moreover, despite a growing interest in transgenerational effects and epigenetic inheritance, only very little is known about the role of paternal behaviour in shaping offspring behaviour outside the research areas of complex diseases and the study of human psychobiology [28]. In the latter, the interaction between environmental and genetic factors can affect the development of antisocial behaviour [29] and the risk of developing stress-induced disorders [30]. Recent studies on chickens (*Gallus gallus*) indicate transgenerational epigenetic inheritance of parental stress, as gene expression patterns were found to correlate between parents and their sons [31] or offspring generally [32]. However, studies attempting to disentangle relatively stable behavioural traits such as personality traits from plastic, short-term environmentally induced epigenetic effects are currently scarce.

In the present study, we aimed to assess the relative importance of personality traits presumed to have a strong genetic basis and potential epigenetic inheritance induced by manipulations of social status of fathers for shaping offspring activity levels in the zebrafish. Offspring activity levels are likely to affect offspring fitness when searching for food sources and may therefore be indicative of exploratory behaviour, with more explorative individuals being more likely to exploit novel food patches (as shown in [33]). We assessed males repeatedly for their exploratory behaviour (boldness) and then forced them to change their social status. Following the experimental treatments, we performed in vitro fertilisations (IVFs) using a split-cross design and monitored the activity levels of the resulting offspring during early life. We found strong effects of stable paternal personality traits as well as experimentally induced paternal social status effects on offspring activity, which seems to indicate that both genetic factors as well as epigenetic effects are involved in shaping offspring behaviour.

## Methods

### Study species

The zebrafish used in this experiment were outbred AB wildtype descendants of fish purchased at ZIRC (Zebrafish International Resource Center, University of Oregon, Eugene, USA) that had been raised to maturity under standard laboratory conditions in the SciLifeLab facilities at the Evolutionary Biology Center at Uppsala University. The facilities feature a 12 h:12 h dark: light regime, and a constant temperature of 28 °C. The fish used in the current study were 4–7 months old adults. All fish were fed ad libitum twice per day, with dried flake food in the morning and live artemia larvae in the afternoon. The Swedish Ethical standards were respected and all experimentation approved (Jordbruksverket Approval No C341/11). The exact relatedness among experimental fish is unknown, but inbreeding is assumed to be low due to large population sizes in the stock tanks, the careful breeding design, random pairing, and employment of a split-clutch design.

### Male behaviour

**Fig. 1:**
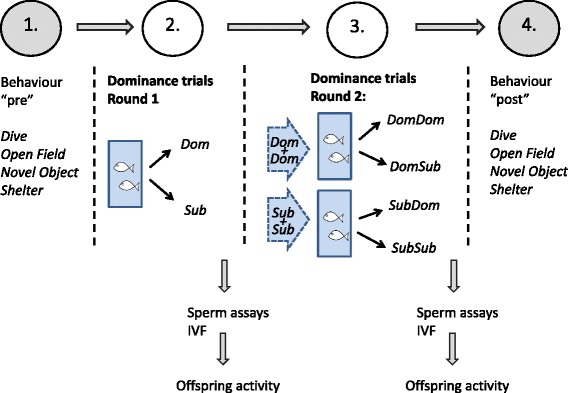
Study design and flow of experiments for each male. Behavioural personality assays (for details see Additional file 1) were conducted before and after all other trials. Social rank manipulations took part in two rounds, each of which was followed by sperm assays, in-vitro fertilisations, and subsequent offspring activity assays

To explore the relationship and consistency in the personality of males with alterations in social status, we repeatedly tested experimental males in two series of behavioural assays. One series of personality assays was completed before tests of social status began, and another after all tests had been concluded (approximately four weeks later, see Fig. 1). We chose four different assays (a 5 min dive assay, and open field, novel object and shelter assays lasting 10 mins each, see Supp. Mat. for details), which measured the fish’s response to novelty, exploration behaviour and timidity, which are all indicative of personality [13]. Animals were expected to show individual variation in personality along a bold-shy axis [34]. Males were assigned to these assays in random order, with no fish performing more than one trial per day. All four personality assays were completed within one week for each fish.

To investigate the consistency of the behaviour over time, as well as its context-dependence [35], the four assays were initially done on *N* = 48 males. However, only *N* = 36 males produced offspring twice (see Fig. 1 for timing of the different experimental components), and therefore males that did not produce any offspring either in Round 1 or 2 were excluded.

### Social status trials

All experimental males were exposed to two consecutive experimental rounds where we manipulated the social status of males (Fig. 1). Males were uniquely colour coded using VIE tags (Visible Implant Elastomer Tags, NMT INC Northwest Marine Technology, Shaw Island, WA, USA) several weeks before the start of the experiment. In the first round (Round 1), two size-matched males (body length ± no more than 1 mm, i.e. less than 5% difference between the pair) were put into a three-litre tank for seven days in order to form a social hierarchy. Each tank contained plastic plants for spatial heterogeneity and to provide hiding space for the subordinate individual. Zebrafish form hierarchies when kept in small groups that do not allow the formation of schools [27]. The fish were observed three times per day for seven days and scored for signs of dominance (chasing, biting, foraging) and subordination (hovering in corners, on bottom or near surface, hiding, fleeing), in trials lasting 10 mins per male-male pair per time point [27]. Usually within 24 h but at the latest within 48 h, stable relationships between dominant and subordinate fish had formed. After seven days, sperm samples from both males in each tank were taken for sperm measurements and in vitro fertilisations (see below for more details). The fish were left to recover on their own for 24 h.

In the second round (Round 2), males were recombined and reallocated to tanks in pairs with males of equal social status as acquired in the first seven days of the experiment. To do so, we combined size-matching dominant males with dominant males, and subordinate males with subordinate males of equal size, which forced one dominant male per tank to become subordinate and vice versa. These pairings were again kept for seven days, after which sperm samples were collected for sperm measurements and in vitro fertilisations. Due to handling constraints, the experiment took place in three blocks.

### Gamete collection and in vitro fertilizations

To collect and process sperm and egg samples, we followed the procedures described in Zajitschek et al. ([12], see Supp. Mat.). In total, we obtained *N* = 132 clutches from 65 females (73 clutches from 35 females after the Round 1, 59 clutches from 31 females after Round 2). We used a split-clutch design, where each male fertilised eggs from two different females at each Round, and with the aim of having each female’s eggs fertilised by two males. However, splitting depended on total number of eggs obtained per female, which varied between *N* = 18 (in which case the clutch was not split), and *N* = 150 (in which case the clutch was split in 8 parts). In total, *N* = 94 of the 132 (sub-)clutches were successfully fertilized and produced viable offspring, resulting in a total of *N* = 1399 offspring.

### Offspring activity assays

We checked for successful fertilisation one hour post fertilisation (pf), and allocated up to 12 individual offspring per male-female pairing to cells within 6-well culture plates. Excess offspring from large clutches were humanely euthanised. The locations in the culture plates were individually marked and the plates covered before incubation at 28 °C. Because zebrafish larvae initially live of their large yolk provisions and only transition into the free-swimming and foraging stage at approximately day nine pf [36], we chose seven and ten days pf as two points in time at which we predicted to detect changes in activity patterns. The offspring were filmed at each time point for at least 10mins using a Sony DCR-SR32E Handycam. Experiments were also done at 28 °C ambient room temperature. We used semi-automated tracking software (CTrax v 0.2.1;, [37]) to record the x,y positions of each fish within the culture plates. Any software errors were manually corrected with the associated FixErrors GUI software package in MATLAB (2011). From these trajectories, we calculated each fish’s median speed (in mm per second) across each trial as a measure of their activity [38]. We assumed that activity is a relevant trait, as it a) may reflect exploratory behavior and hence ability to exploit suitable feeding grounds after hatching and b) may be indicative of future boldness. Due to small changes in the lighting conditions in some of the videos disrupting the tracking process, the length of tracks differed between videos (see Additional file 1: Figure S1A; Mean time: 9.59 min ± 0.07 SE, at 25 frames per second). However, we checked that the number of frames we tracked the fish for, and the time at which we started the tracking was not related to the fish’s activity.

### Statistical analyses

We performed a principal component analysis including all behavioural traits measured in the four personality assays (consisting of six individual measures in each assay, which included for example latency to start moving, duration of movement, number of freezing bouts, number of times a threshold was reached; depending on the respective assay. *N* = 24) using the Bioconductor/biocLite package “pcaMethods” [39]. The first principal component (PC) across the behaviour before the experiment (PC1pre) explained 40% of the variation (see Additional file 1: Table S1 for details). PC2pre explained 11%, PC3pre explained 9% and PC4pre to PC20pre explained together the remaining 40%.

Analyses to test for an effect of paternal behaviour and social status on sperm traits (ejaculate volume, density, sperm longevity, VCL (curvilinear velocity), VAP (average path velocity) and VSL (straight line velocity)) and offspring behaviour were performed using linear mixed models (lme4 package for R, v.1.1–9, [40], lmerTest package for R, v.2.0–29, [41, 42]), and meeting of model assumptions visually confirmed. All analyses were conducted using R version 2.15.3 [42]. Social status was treated as a two-level factor (dominant: Dom, subordinate: Sub) in analyses on round A and when combining data for both rounds. In analyses on round B only, we used four different levels to reflect the history of each male (i.e. DomDom – DomSub – SubDom – SubSub). The models investigating offspring activity contained the information on male social status (MSS), male personality (PC1pre) and offspring age (Age), and all interaction terms of these factors. Models investigating the association between offspring activity and sperm swimming velocity (VCL) included full interaction terms of VCL with male personality (PC1pre), male social status in Round 1 (MSSA) and Round 2 (MSSB), as well as offspring age at trial (Age). Interaction terms were retained due to significance at 0.05 level. In all analyses, parental IDs (i.e. male and female identifiers) were included as random variables. All analyses presented below were performed including those males for which IVFs could be successfully performed and offspring activity could be collected after *both* experimental rounds (*N* = 23) [43].

## Results

### Male behavioural traits

Behavioural traits were found to be highly repeatable (average intraclass-correlation coefficient ICC = 0.708, CI_lower_ = 0.636. CI_upper_ = 0.767 in males that maintained dominance status, ICC = 0.691 CI_lower_ = 0.634. CI_upper_ = 0.738 across all males (regardless of dominance status switches or not), ICC = 0.666, CI_lower_ = 0.570. CI_upper_ = 0.741 in males that switched their dominance status only). We found no evidence that male social status (MSS) was related to male behaviour, regardless if tested on raw behavioural responses or PCs, and irrespective of the round of dominance trials or the timing of personality assays. The first PC across all behavioural trials before the experiment (PC1pre) largely coded for behaviour along the bold-shy continuum, whereas the second PC may be indicative of activity (Additional file 1: Table S1). We included behaviour and MSS as independent variables in our models to test the effects of personality on sperm traits and on activity patterns in the next generation.

### Sperm traits

**Fig. 2:**
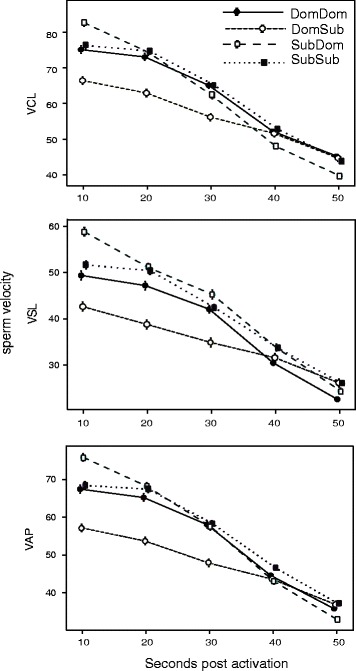
Sperm velocity declined (seconds post activation) in three measures of velocity (VCL, curvilinear, VSL: straight-line, VAP: average path) after males had completed Round 2 of dominance trials. Males forced into subordinate (Sub) roles after being dominant (Dom) showed lower initial sperm velocity

Neither MSS nor behaviour explained variation in ejaculate volume, density or motility (percentage of non-motile sperm), at any time point. Sperm longevity (seconds of forward motility since activation), however, was influenced by behaviour (Table S2). Sperm velocity was strongly affected by MSS both after completion of Round 1 as well as Round 2. After Round 1, dominant males exhibited slower initial sperm velocities than subordinate males, but their rate of decline over time was lower than in subordinate individuals. Males that started out as dominant in Round 1 and became subordinate in Round 2 (DomSub males) produced significantly slower swimming sperm with velocity declining less rapidly than all other males (Fig. 2). In addition, male behaviour (PC2pre) showed significant interaction terms on all velocity traits (curvilinear velocity, VCL; straight-line velocity, VSL; average path velocity VAP, Additional file 1: Table S3).

**Fig. 3:**
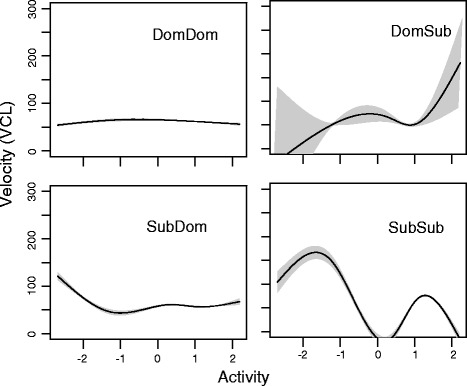
Example for the influence of behaviour on sperm velocity (Curvilinear velocity, VCL) at 30 s post activation. “Activity” is derived from interpreting the second principal component (see Additional file 1: Table S1)

To visualize the complexity of the interactions between behaviour and social status on sperm (Fig. 3), we used general additive mixed models (GAMMs) in the package MGCV 1.7–29 to model the behavioural component that was identified as the most influential for sperm velocity after two rounds of dominance trials (PC2pre) in its relationship to VCL. VCL is the most ecologically meaningful trait in zebrafish sperm velocity, but all velocity measures were highly correlated (VAP-VCL: *r* = 0.97; VSL-VCL: *r* = 0.86; VSL-VAP: *r* = 0.92, see also Fig. 2). VCL was measured during 30 s post activation, which was arbitrarily chosen because the influence of the behavioural variable on the observed pattern stayed the same, while velocity itself declined over time. This indicated that the most active dominant fish that subsequently became subordinate had higher sperm activity than less active individuals within this group.

### Offspring traits

**Fig. 4:**
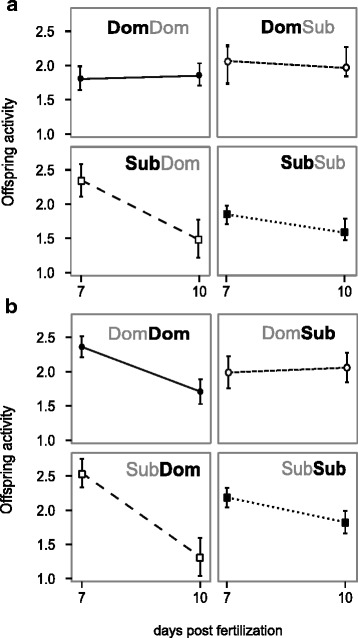
Offspring activity at 7 and 10 days post fertilization in response to male social status. **a** After Round 1, offspring of dominant males (Dom) were more active overall, whereas offspring from subordinate males (Sub) showed a drop in activity from day 7 to day 10. **b** After Round 2, activity patterns in offspring were similar across the four groups except for offspring sired by males which were dominant in both rounds. In this group, offspring activity dropped significantly from day 7 to day 10. Dominance status shown in black indicates the status held in the current trial, whereas dominance status shown in *grey* indicates the status held in the previous or later trial

Social status, behavioural traits and their interactions were significantly associated with offspring activity after both, the first as well as the second round (Table 1), indicating an influence of both paternal personality (assumed to be relatively stable) as well as experimentally manipulated paternal social status (indicating highly plastic, potentially epigenetically induced effects) on offspring activity patterns. The effect of both behaviour and social status and their interactions were more pronounced after Round 2 had been completed, i.e. after some individuals had undergone a dominance status shift. After Round 1, we found that offspring activity was consistent across the two age classes in the offspring of dominant males, but dropped from day 7 to day 10 in offspring from subordinate males (Table 1a; Fig. 4a). After Round 2, offspring activity patterns were similar for the dominant-subordinate, subordinate-dominant and subordinate-subordinate MSS, whereas the activity pattern of offspring from males exhibiting a dominant-dominant status changed considerably (Table 1b; Fig. 4b). Males that maintained their dominance status between rounds had offspring that had lower activity after 10 days compared to their offspring that were tested before the dominance trials. Analysing the data of two rounds jointly confirms the strong interaction between MSS during both rounds and male behaviour (Table 1C). We also found a significant association between offspring activity and sperm velocity (Table 2).

**Table 1:** Effects of male behaviour and male social status on offspring activity after Round 1 (A, *N* = 395 offspring) and Round 2 (B, *N* = 611 offspring) of the experiment and across both rounds combined (C). Statistical parameters come from a linear mixed model (REML) with a Type II Wald *Χ* ^*2*^ test. Parental IDs are included as random variables in all models

	Χ^2^	df	*P*
A			
Age	0.28	1	0.60
Male behaviour (PC1pre)	2.03	1	0.15
Male social status (MSS)	2.28	1	0.52
Age x PC1pre	3.45	1	0.06
Age x MSS	2.82	1	0.42
PC1pre x MSS	16.32	1	0.001*
Age xPC1pre x MSS	15.30	1	0.002*
B			
Age	18.72	1	<0.001*
Male behaviour (PC1pre)	20.74	1	<0.001*
Male social status (MSS)	12.83	3	0.005*
Age x PC1pre	20.31	1	<0.001*
Age x MSS	12.09	3	0.007*
PC1pre x MSS	23.35	3	<0.001*
Age x PC1pre x MSS	23.62	3	<0.001*
C			
Age	11.61	1	<0.001*
Behaviour (PC1pre)	27.51	1	<0.001*
Social status A (MSSA)	0.09	1	0.77
Social status B (MSSB)	2.69	1	0.10
MSSA x MSSB	2.71	1	0.099
Age x PC1pre	29.74	1	<0.001*
Age x MSSA	0.25	1	0.62
Age x MSSB	3.31	1	0.13
PC1pre x MSSA	0.40	1	0.53
PC1pre x MSSB	0.08	1	0.78
Age x MSSA x MSSB	3.25		0.07
Age x PC1pre x MSSA	0.28	1	0.60
Age x PC1pre x MSSB	0.20	1	0.66
PC1pre x MSSA x MSSB	5.65	1	0.02*
Age x PC1pre x MSSA x MSSB	5.63	1	0.02*

* indicates significance (α < 0.05)

**Table 2:** Significant association between offspring activity and sperm swimming velocity (VCL) across both rounds combined. Statistical parameters come from a linear mixed model (REML) with a Type II Wald *Χ* ^*2*^ test. Male and female IDs are included as random variables

	*Χ* ^*2*^	df	*P*
Age	16.66	1	<0.001*
Behaviour (PC1pre)	36.46	1	<0.001*
Social status A (MSSA)	0.12	1	0.73
Social status B (MSSB)	0.91	1	0.34
VCL	12.40	1	<0.001*
PC1pre x Age	38.34	1	<0.001*
MSSA x Age	0.04	1	0.85
MSSB x Age	1.23	1	0.27
VCL x Age	13.78	1	<0.001*
PC1pre x VCL	0.08	1	0.78
PC1pre x VCL X Age	75.61	1	<0.001*

* indicates significance (α < 0.05)

## Discussion

Zebrafish offspring activity appears to be affected by interactions between relatively stable, putatively genetic and plastic, epigenetic factors of paternal behaviour where both, behaviour associated with paternal boldness and social status influenced offspring activity. In particular, males starting off as dominant and being subordinate in the second round showed the strongest effects on ejaculate traits as well as offspring activity, as their sperm was consistently slower than the sperm of all other males, but their offspring maintained a higher activity at the age of ten days pf after the second round. Males that maintained a dominant status during both experimental rounds showed the strongest shift in offspring activity when compared across the two rounds, whereas their sperm traits were similar to those of males being subordinate across both rounds or switching from subordinate to dominant. Overall, our results suggest that there is an interaction between parental genetic and non-genetic factors that determine offspring behaviour.

Interestingly, we found no significant relationship between paternal boldness and dominance rank, even in males that maintained their social status throughout the experiment. This finding is somewhat surprising given the fact that these relationships have been documented in zebrafish before [23], but also in other species including the rainbowfish *Melanotaenia duboulayi* [44] and the zebrafinch *Taeniopygia guttata* [45]. Our results may indicate that social status and boldness are not necessarily as strongly coupled as previously assumed. Differences between studies may arise due to variation in experimental design. In fact, the previous study in zebrafish showing that boldness could be predicted based on their social status was assessed in individuals of both sexes across three different assays without testing for repeatability [23]. Furthermore, the variables analysed differed somewhat between this previous study and included distance moved, but no variable on freezing behaviour, which we found to be an important indicator of anxiety and shyness. These discrepancies across different studies confirm that we still have very little understanding of the heritability and consistency of behavioural patterns. Nevertheless, our results support the theoretical predictions that personality traits are context-dependent and are consistent with findings in a range of other taxa [19, 20, 25]. A study in the domestic fowl *Gallus gallus domesticus* showed that current social status contributes critically to both variation and stability in behavioural responses [22]. Similarly, social context was found to influence behaviour in mink *Neovison vison,* despite generally stable behavioural responses in repeated trials [46]. Furthermore, in the African cichlid *Oreochromis mossambicus,* behavioural responses were inconsistent over time and largely depended on the social context [47].

The environmental context, both social and ecological, can play a major role for the expression of behavioural phenotypes [20]. The social context has recently been highlighted in affecting the expression of personality in group living animals, and plays fundamental roles for individual behaviour as well as in group dynamics (reviewed in [48]). In fact, even short-term social circumstances and experiences can have profound effects on behavioural performance. Bystander effects, where changes in behaviour are the result of watching the performance of conspecifics, influence the level of boldness in rainbow trout *Onchorhyncus mykiss* [49]. Similarly, ecological conditions may play an important role in the manifestation of personality. The introduction of predation pressure can induce a correlation between boldness and aggression, which is absent under low predation pressure in threespined stickleback, *Gasterosteus aculeatus* [50]. The correlation between proportion of extra-pair paternity and response to novel objects also varies according to operational sex ratio in a large-scale study on captive zebra finches *Taeniopygia guttata* [24]. These findings emphasize the context-dependence of behavioural traits and the importance of taking the context into account when performing behavioural studies.

Social circumstances are known to not only influence behavioural traits but also affect male physiology and as a result ejaculate traits such as sperm number [51], sperm velocity ([12],e.g. [52, 53]) and sperm morphometry [54, 55]. These traits are generally assumed to increase a male’s success during sperm competition and result in higher reproductive success. However, the fitness advantage may not only be due to increased fertilisation success, but also due to increased fitness in offspring of better competitors [56, 57]. In fact, we found a significant link between sperm swimming velocity and offspring activity. In our study, the decline of sperm velocity over time was markedly different in males that switched from dominant to subordinate status (DomSub males), but not in all other males. In addition, DomSub males that were classified as particularly bold in the novel object trial had faster swimming sperm. This pattern was directly reflected in offspring activity: offspring sired by DomSub males experienced no drop in activity levels between day seven and day ten, whereas offspring sired by all other males did show a drop in activity levels. It is possible that offspring activity is associated with personality and could potentially be predictive of future social status. This could be an interesting area of future research.

The evidence for direct links between offspring traits and sperm-mediated epigenetic effects is mounting rapidly. In particular, the long-term transgenerational effects of maternal exposure to toxins such as vinclozolin on offspring traits in rats *Rattus norvegicus* have been linked to epigenetically induced changes in sperm methylation patterns across multiple subsequent generations (for example [58, 59]). However, so far only few studies provided evidence for the effects of short-term exposure to less toxic treatments, which influence not only the male ejaculate but also offspring performance. In *Drosophila melanogaster* for example, a two-day short-term sugar treatment in fathers affected offspring metabolic state and obesity, and was connected with chromatin-state alterations in sperm [60]. In the red flour beetle *Tribolium castaneum*, transgenerational immune priming was not only linked to paternal transmission, but also indicated transfer via sperm [61]. Our recent study in zebrafish demonstrated the link between the social environment, specifically the level of male-male competition for access to females, sperm velocity, and offspring hatching speed and survival [12].

## Conclusion

Overall, our results highlight that the transgenerational transmission of behavioural patterns is highly complex and context-dependent as predicted by theory [35]. Both the artificially altered social rank as well as the stable paternal personality behaviour had a significant influence on sperm performance and offspring activity. Our findings emphasise the importance to direct our focus on both, genetic and epigenetic components of behaviour in order to fully understand the heritability of behavioural traits.

## Additional file

Additional file 1: Supplemental Material.
**Methods:** 1. Personality assays. 2. Sperm measurements. 3. In vitro fertilisation. **Figure S1:** The number of minutes the fish were tracked for was not related to the activity measures. (A) There was no relationship between number of frames fish were tracked and their mean median speed (Spearman Correlation: *r* = −0.05, *p* = 0.59). (B) There was also no relationship between the mean median speed of fish in a trial, and the time when the tracking begun (Spearman Correlation: *r* = 0.08, *p* = 0.41). **Table S1:** The loadings of the first three principal components for each of the different behavioural trials and their explanatory power. PC1pre for all trials can be interpreted along the bold-shy continuum (indicated by loadings in bold). *“Time to start” refers to the time an individual took to cross a threshold line for the first time (Dive, Novel Object, Open Field) or exit the shelter for the first time (Shelter). “# in centre” / “time in centre” represent the number of times / amount of time a focal male spent within the area of a specified threshold. “# freezing bouts” and Time immobile refer to anxious behaviour, where sudden erratic movements and remaining entirely still may alternate. **Table S2:** Sperm longevity (in seconds since activation), ejaculate volume (μl) and motility (% of motile sperm cells), after the first round of dominance trials and after the second (taking the social status of the first round into account, MSS). Interaction terms were not significant and were hence not included. Degrees of Freedom are based on Satterthwaite approximations for type III ANOVA. **Table S3**: Effects of male behaviour and male social status on sperm velocity measures VCL (curvilinear velocity); VSL (straight-line velocity); VAP (average path velocity) after round 2. “Time” indicates the decline in velocity measured in 10 s steps since activation, “Time^2^” investigates the curvature in the decline. Significant explanatory variables are highlighted by an asterisk (*). (DOCX 101 kb)

## Abbreviations

Dom: Dominant social status
Domdom: Dominant social status in both rounds of trials
DomSub: Changed social status from dominant in the first round to subordinate in second round
GAMMs: General additive mixed models
ICC: Intraclass-correlation coefficient
IVF: in-vitro fertilisation
MSS: Male social status
PC: Principal component
PC1pre (PC2pre, etc): First (second, etc) principal component across the behavioural trials that took place before the experiment
Pf: Post fertilisation
Sub: Subordinate social status
SubDom: Changes social status from subordinate in the first round to subordinate in the second round.
SubSub: Maintained subordinate social status across both trials
Supp. Mat: Supplementary material
VAP: Average path velocity
VCL: Curvilinear velocity
VIE tags: VISIBLE Implant Elastomer Tags
VSL: Straight-line velocity
ZIRC: Zebrafish International Resource Center
