# Supplementary material for: Paternal personality and social status influence offspring activity in zebrafish
Source: BMC Evol Biol. 2017 Jul 3;17:157. doi: 10.1186/s12862-017-1005-0 (PMC5496241; doi:10.1186/s12862-017-1005-0)
Supplement: Additional file 1: Supplemental Material. — Methods: 1. Personality assays. 2. Sperm measurements. 3. In vitro fertilisation. Figure S1: The number of minutes the fish were tracked for was not related to the activity measures. (A) There was no relationship between number of frames fish were tracked and their mean median speed (Spearman Correlation: r = −0.05, p = 0.59). (B) There was also no relationship between the mean median speed of fish in a trial, and the time when the tracking begun (Spearman Correlation: r = 0.08, p = 0.41). Table S1: The loadings of the first three principal components for each of the different behavioural trials and their explanatory power. PC1pre for all trials can be interpreted along the bold-shy continuum (indicated by loadings in bold). *“Time to start” refers to the time an individual took to cross a threshold line for the first time (Dive, Novel Object, Open Field) or exit the shelter for the first time (Shelter). “# in centre” / “time in centre” represent the number of times / amount of time a focal male spent within the area of a specified threshold. “# freezing bouts” and Time immobile refer to anxious behaviour, where sudden erratic movements and remaining entirely still may alternate. Table S2: Sperm longevity (in seconds since activation), ejaculate volume (μl) and motility (% of motile sperm cells), after the first round of dominance trials and after the second (taking the social status of the first round into account, MSS). Interaction terms were not significant and were hence not included. Degrees of Freedom are based on Satterthwaite approximations for type III ANOVA. Table S3: Effects of male behaviour and male social status on sperm velocity measures VCL (curvilinear velocity); VSL (straight-line velocity); VAP (average path velocity) after round 2. “Time” indicates the decline in velocity measured in 10 s steps since activation, “Time2” investigates the curvature in the decline. Significant explanatory variables are highlighted by an asterisk (*). (DOCX 101 kb) [file 12862_2017_1005_MOESM1_ESM.docx]

The influence of paternal personality and social status on offspring activity in zebrafish

# Susanne Zajitschek^1,2*^, James Herbert-Read^3^, Nasir Abbasi^1^, Felix Zajitschek^4^ & Simone Immler^1^

1 Department of Ecology and Genetics, Evolutionary Biology, Uppsala University, Norbyvägen 18D, SE-752 36 Uppsala, Sweden

2 Doñana Biological Station EBD-CSIC, C/ Americo Vespucio s/n, 41092 Isla de la Cartuja,

Sevilla, Spain

3 Department of Mathematics, Uppsala University, Lägerhyddsvägen 1, 751 06 Uppsala, Sweden

4 School of Biological Sciences, Monash University, Building 18, Victoria 3800, Australia

**Supplemental Material**

**Methods**

*1. Personality assays*

*Assay 1: DIVE*. In a five-minute dive test (Cachat et al., 2010), males were transferred into narrow 1.5 litre tanks with a drawn-on line indicating half-height of the water level (located approximately 7cm from the bottom of the tank). Fish newly transferred to these tanks typically hover near the ground. We recorded the latency to start moving, the time until the fish first crossed into the upper part of the tank, the number of times the line was crossed while exploring the tank, the number of erratic movements and the duration and number of freezing bouts.

*Assay 2: OPEN FIELD.* In an open field test (see for example Dahlbom et al., 2011; Stewart et al., 2010), the fish spent fifteen minutes in a custom build circular plastic tank (40 cm diameter/ height 10 cm) with opaque side walls and a clear Perspex bottom. A centrally aligned paper with an r=12 cm ring representing the border between “outside of arena” and “inside of arena”, was placed underneath the tank. We observed on latency to first moving, time spent inside the arena, and the number of times crossing from the outside into the inside area. The number and duration of freezing bouts and erratic movements as well as total time spent swimming were recorded. Each trial lasted ten minutes and was started after a five-minute acclimation time, which was given to allow the fish to recover from handling stress and respond to the novel environment rather than to fear due to handling.

*Assay 3*: *NOVEL OBJECT.* In the novel object assays, a red and a blue Lego® brick were stacked together and placed in the centre of the arena before the introduction of the fish. After a five-minute acclimation period, the same behaviour as described for the open field trials was recorded for ten minutes. The novel object assays also took place in the round custom tank described in Trial 2.

*Assay 4*: *SHELTER*. In the shelter assays, a plastic box with a remotely opening door (pulled up via a string) was inserted near the edge of the round observation arena (same type of tanks as in the open field and novel object assays). The focal male was introduced into this box and after five minutes the door was carefully lifted. We recorded the latency to exit the shelter, the time spent outside the shelter as well as the number of instances returning into the shelter, for a total of ten minutes.

*2. Sperm measurements*

To collect sperm samples, we followed the procedures described in Zajitschek et al. (2014). In brief, anaesthetized males were gently stroked several times in a cranio-caudal direction using smooth forceps. The sperm appearing at the genital pore was collected with a microcapillary and stored in 20 µl of Hank’s solution (HBSS) until examination (60-120 minutes post collection).

One µl of the sperm solution was placed on a 20 μm depth glass cell counting chamber (2X-CEL Chamber, Hamilton-Thorne), covered with a cover slip and activated with 5 µl of tank water. Sperm velocity was recorded using CASA software (ISAS Proiser, Valencia, Spain). Velocity recordings were taken during one second in 10 second intervals (starting 10s post activation) and continued until > 95% of the sperm had stopped moving (50 - 80s), using the following settings: frame rate: 50 frames per second, frames used: 50, particle area: 5-50 μm^2^, threshold measurements for VCL values (μm / second): 10 < slow < 45 < medium < 100 < rapid. Three to four replicate velocity measurements were taken for each sample. For the velocity analyses, slow and static sperm were excluded. The samples used in the analyses contained on average 265.1 motile sperm (range 112 -1918), 10 seconds post activation.

*3. In vitro fertilisation*

After the collection of sperm, we collected eggs with gentle abdominal massage from females following the protocol described in Zajitschek et al. (2014). We obtained egg clutches from females that had been kept under standard conditions in groups of 16 fish with 1:1 sex ratio for two weeks. Females were anaesthetized, rinsed, and lightly dry-blotted and placed into a 35 mm Petrie dish. Eggs were obtained by gentle squeezing along the sides of the body towards the genital opening with damp finger tips. Any contact of the eggs with water was carefully avoided prior to fertilisation in order to avoid premature egg activation. The female was revived in warm tank water straight after the stripping procedure. We aimed to use approximately 15 eggs for a given fertilisation set. Therefore, large clutches were split according to the numbers of viable eggs (yellowish, translucent) obtained from each female. In total, we obtained N= 132 clutches from 65 females (73 clutches from 35 females after the first dominance trial, 59 clutches from 31 females after the second trial). Subdivided clutches were placed in separate Petrie dishes and each sub-clutch fertilized with sperm from different males, by adding 7 µl of sperm solution and 14 µl of tank water for activation to each clutch. Each male’s sperm was used to fertilize sub-clutches from two different females at each IVF time point (i.e. after the first and after the second dominance periods, respectively). This “split-clutch” design allowed us to investigate the paternal effects on offspring development while also controlling for maternal effects. In total, N=94 of the 132 (sub-)clutches were successfully fertilized and produced viable offspring (58% success after the first Dominance trial, 88% success after the second round; the low fertilisation success in the first round was most likely due to sub-optimal egg quality), resulting in a total of N=1399 offspring.

**Figure S1**: The number of minutes the fish were tracked for was not related to the activity measures. (A) There was no relationship between number of frames fish were tracked and their mean median speed (Spearman Correlation: r = -0.05, p = 0.59). (B) There was also no relationship between the mean median speed of fish in a trial, and the time when the tracking begun (Spearman Correlation: r = 0.08, p = 0.41).

***References cited***

Cachat, J., Stewart, A., Grossman, L., Gaikwad, S., Kadri, F., Chung, K.M., Wu, N., Wong, K., Roy, S., Suciu, C., et al. (2010). Measuring behavioral and endocrine responses to novelty stress in adult zebrafish. Nat Protoc. *5*, 1786–1799.

Dahlbom, S.J., Lagman, D., Lundstedt-Enkel, K., Sundstrom, L.F., and Winberg, S. (2011). Boldness Predicts Social Status in Zebrafish (Danio rerio). Plos One *6*, 7.

Stewart, A., Cachat, J., Wong, K., Gaikwad, S., Gilder, T., DiLeo, J., Chang, K., Utterback, E., and Kalueff, A.V. (2010). Homebase behavior of zebrafish in novelty-based paradigms. Behav. Processes *85*, 198–203.

Zajitschek, S., Hotzy, C., Zajitschek, F., and Immler, S. (2014). Short-term variation in sperm competition causes sperm-mediated epigenetic effects on early offspring performance in the zebrafish. Proc. R. Soc. Lond. B Biol. Sci. *281*.

**Supplementary Tables**

**Table S1:** The loadings of the first three principal components for each of the different behavioural trials and their explanatory power. PC1pre for all trials can be interpreted along the bold-shy continuum (indicated by loadings in bold).

*“Time to start” refers to the time an individual took to cross a threshold line for the first time (Dive, Novel Object, Open Field) or exit the shelter for the first time (Shelter). “# in centre” / “time in centre” represent the number of times / amount of time a focal male spent within the area of a specified threshold. “# freezing bouts” and Time immobile refer to anxious behaviour, where sudden erratic movements and remaining entirely still may alternate.

| **Assay** | **Behaviour*** | **PC1pre** | **PC2pre** | **PC3pre** |
| --- | --- | --- | --- | --- |
| \| Dive \| \| --- \| \|  \| \|  \| \|  \| \|  \| \| Novel Object \| \|  \| \|  \| \|  \| \|  \| \| Open Field \| \|  \| \|  \| \|  \| \|  \| \| Shelter \| \|  \| \|  \| \|  \| \|  \| | \| Time to start \| \| --- \| \| # in centre \| \| Time in centre \| \| # freezing bouts \| \| Time immobile \| \| Time to start \| \| # in centre \| \| Time in centre \| \| # freezing bouts \| \| Time immobile \| \| Time to start \| \| # in centre \| \| Time in centre \| \| # freezing bouts \| \| Time immobile \| \| Time to exit \| \| # in centre \| \| Time in centre \| \| # freezing bouts \| \| Time immobile \| | \| **-0.286** \| \| --- \| \| **0.317** \| \| **0.304** \| \| -0.177 \| \| -0.130 \| \| -0.133 \| \| **0.283** \| \| **0.303** \| \| -0.114 \| \| -0.198 \| \| -0.162 \| \| **0.291** \| \| **0.257** \| \| -0.173 \| \| -0.235 \| \| -0.035 \| \| **0.269** \| \| 0.147 \| \| -0.116 \| \| **-0.254** \| | \| 0.158 \| \| --- \| \| 0.052 \| \| 0.115 \| \| 0.381 \| \| 0.318 \| \| **-0.412** \| \| 0.194 \| \| 0.171 \| \| -0.118 \| \| -0.004 \| \| 0.233 \| \| 0.020 \| \| 0.068 \| \| -0.042 \| \| 0.128 \| \| 0.210 \| \| -0.037 \| \| -0.256 \| \| **-0.500** \| \| 0.152 \| | \| -0.213 \| \| --- \| \| 0.116 \| \| 0.171 \| \| -0.090 \| \| -0.427 \| \| -0.210 \| \| 0.242 \| \| 0.026 \| \| 0.296 \| \| 0.099 \| \| 0.224 \| \| -0.179 \| \| -0.184 \| \| 0.417 \| \| 0.074 \| \| 0.448 \| \| -0.128 \| \| 0.027 \| \| 0.078 \| \| -0.034 \| |
|  | **Proportion of**  **variance** | **0.40** | **0.11** | **0. 09** |

**Table S2:** Sperm longevity (in seconds since activation), ejaculate volume (µl) and motility (% of motile sperm cells), after the first round of dominance trials and after the second (taking the social status of the first round into account, MSS). Interaction terms were not significant and were hence not included. Degrees of Freedom are based on Satterthwaite approximations for type III ANOVA

|  | **Sperm longevity** | | | | **Ejaculate size** | | | **Sperm motility** | | |
| --- | --- | --- | --- | --- | --- | --- | --- | --- | --- | --- |
| ***First round*** | ***df*** | ***F*** | | ***p*** | ***df*** | ***F*** | ***p*** | ***df*** | ***F*** | ***p*** |
| MSS | *1,18* | 0.000 | 0.988 | | *1,18* | 0.807 | 0381 | *1,17* | 0.799 | 0.384 |
| PC1pre | *1,18* | 5.397 | **0.032** | | *1,18* | 3.127 | 0.094 | *1,19* | 0.628 | 0.438 |
| PC2pre | *1,18* | 1.860 | 0.189 | | *1,18* | 2.513 | 0.130 | *1,16* | 1.056 | 0.319 |
| PC3pre | *1,18* | 1.262 | 0.276 | | *1,18* | 0.000 | 0.994 | *1,18* | 0.009 | 0.925 |
| ***Second round*** |  |  |  | |  |  |  |  |  |  |
| MSS | *3,13* | 0.160 | 0.921 | | *3,13* | 0.245 | 0.863 | *3,12* | 0.958 | 0.443 |
| PC1pre | *1,13* | 0.330 | 0.576 | | *1,13* | 0.003 | 0.956 | *1,15* | 0.018 | 0.895 |
| PC2pre | *1,13* | 12.004 | **0.004** | | *1,13* | 3.423 | 0.087 | *1,13* | 0.012 | 0.913 |
| PC3pre | *1,13* | 2.051 | 0176 | | *1,13* | 2.380 | 0.147 | *1,14* | 0.022 | 0.884 |
| PC1post | *1,13* | 0.047 | 0.832 | | *1,13* | 1.936 | 0.187 | *1,14* | 0.512 | 0.486 |
| PC2post | *1,13* | 6.169 | **0.027** | | *1,13* | 1.848 | 0.197 | *1,12* | 1.874 | 0.197 |
| PC3post | *1,13* | 0.675 | 0.426 | | *1,13* | 0.046 | 0.834 | *1,12* | 0.826 | 0.381 |

Table S3: Effects of male behaviour and male social status on sperm velocity measures VCL (curvilinear velocity); VSL (straight-line velocity); VAP (average path velocity) after round 2. “Time” indicates the decline in velocity measured in 10 sec steps since activation, “Time^2^” investigates the curvature in the decline. Significant explanatory variables are highlighted by an asterisk (*).

|  | **VCL** | | | | | | **VSL VAP** | | | | | |  |
| --- | --- | --- | --- | --- | --- | --- | --- | --- | --- | --- | --- | --- | --- |
|  | ***df*** | | ***t*** | | ***p*** | | ***t*** | | ***p*** | | ***t*** | | ***p*** |
|  | |  |  |  | |  | |  | |  | |  | |
| Male behaviour (PC1pre) | | *1* | -5.115 | **<0.001*** | | -6.142 | | **<0.001*** | | -5.115 | | **<0.001*** | |
| Male social status (MSS) | | *3* |  |  | |  | |  | |  | |  | |
| DomSub | |  | 0.148 | 0.884 | | -0.972 | | 0.343 | | 0.148 | | 0.884 | |
| SubDom | |  | 0.697 | 0.456 | | -0.200 | | 0.844 | | 0.697 | | 0.456 | |
| Sub Sub | |  | -0.106 | 0.913 | | -0.232 | | 0.820 | | -0.106 | | 0.913 | |
| Time | | ***1*** | -12.044 | **<0.001*** | | -7.624 | | **<0.001*** | | -12.044 | | **<0.001*** | |
| Time^2^ | | ***1*** | -2.471 | **<0.001*** | | -5.201 | | **<0.001*** | | -2.471 | | **<0.001*** | |
| PC1pre x MSS | | ***3*** |  |  | |  | |  | |  | |  | |
| PC1pre x DomSub | |  | 1.713 | 0.104 | | 3.093 | | 0.005* | | -5.761 | | **<0.001*** | |
| PC1pre x SubDom | |  | 1.047 | 0.310 | | 1.885 | | 0.075* | | -0.464 | | 0.648 | |
| PC1pre x SubSub | |  | 3.165 | **0.005*** | | 4.513 | | **<0.001*** | | 0.450 | | 0.659 | |
| PC1pre x Time | | ***1*** | 8.559 | **<0.001*** | | 7.526 | | **<0.001*** | | -0.039 | | 0.969 | |
| Time x MSS | | ***3*** |  |  | |  | |  | |  | |  | |
| Time x DomSub | |  | -4.311 | **<0.001*** | | -1.525 | | 0.127 | | -9.012 | | **<0.001*** | |
| Time x SubDom | |  | 1.046 | 0.884 | | 2.185 | | 0.029* | | -6.070 | | **<0.001*** | |
| Time x SubSub | |  | 5.454 | **<0.001*** | | 3.129 | | 0.002* | | 2.528 | | 0.021 | |
| PC1pre x Time^2^ | | ***1*** | -4.568 | **<0.001*** | | -4.871 | | **<0.001*** | | 1.377 | | 0.186 | |
| **(Table S3 continued)** | **VCL** | | | | | | **VSL VAP** | | | | | |  |
|  | ***df*** | | ***t*** | | ***p*** | | ***t*** | | ***p*** | | ***t*** | | ***p*** |
|  | |  |  |  | |  | |  | |  | |  | |
| Time^2^ x MSS | | ***3*** |  |  | |  | |  | |  | |  | |
| Time^2^ x DomSub | |  | 5.118 | **<0.001*** | | 2.718 | | **0.006*** | | 3.831 | | **0.001*** | |
| Time^2^ x SubDom | |  | -0.565 | 0.812 | | -1.527 | | 0.127 | | 8.968 | | **<0.001*** | |
| Time x SubSub | |  | -6.053 | **<0.001*** | | -2.619 | | **0.009*** | | -4.271 | | **<0.001*** | |
| PC1pre x MSS x Time | | ***3*** |  |  | |  | |  | |  | |  | |
| PC1pre x Time x DomSub | |  | -0.237 | 0.812 | | -0.823 | | 0.411 | | -1.171 | | 0.242 | |
| PC1pre x Time x SubDom | |  | -1.938 | 0.053* | | -1.996 | | 0.046 | | -2.540 | | 0.011 | |
| PC1pre x Time x SubSub | |  | -6.470 | **<0.001*** | | -6.093 | | **<0.001*** | | -6.967 | | **<0.001*** | |
| PC1pre x MSS x Time^2^ | | ***3*** |  |  | |  | |  | |  | |  | |
| PC1pre x Time^2^ x DomSub | |  | -1.316 | 0.188 | | -0.264 | | 0.792 | | 0.072 | | 0.943 | |
| PC1pre x Time^2^ x SubDom | |  | 0.839 | 0.401 | | 1.214 | | 0.225 | | 2.013 | | 0.044 | |
| PC1pre x Time^2^ x SubSub | |  | 2.862 | **0.004*** | | 3.300 | | **<0.001*** | | 3.892 | | **<0.001*** | |
